# Aqueous Extract of Brazilian Green Propolis: Primary Components, Evaluation of Inflammation and Wound Healing by Using Subcutaneous Implanted Sponges

**DOI:** 10.1093/ecam/nep112

**Published:** 2011-06-08

**Authors:** Sandra Aparecida Lima de Moura, Giuseppina Negri, Antônio Salatino, Luiza Dias da Cunha Lima, Luana Pereira Antunes Dourado, Juliana Barros Mendes, Silvia Passos Andrade, Mônica Alves Neves Diniz Ferreira, Denise Carmona Cara

**Affiliations:** ^1^Department of General Pathology, Institute of Biological Sciences, Federal University of Minas Gerais-UFMG, Belo Horizonte, Brazil; ^2^CEBRID—Department of Psychobiology, UNIFESP, R. Botucatu, 862, Biomedical Sciences, Brazil; ^3^Department of Botany, Biosciences Institute, University of São Paulo, R. do Matão 277, São Paulo, Brazil; ^4^Department of Physiology, Institute of Biological Sciences, Federal University of Minas Gerais-UFMG, Avenida Antônio Carlos, 6627, Pampulha, CEP 31.270-901, Belo Horizonte, Minas Gerais, Brazil

## Abstract

Propolis is a chemically complex resinous bee product which has gained worldwide popularity as a means to improve health condition and prevent diseases. The main constituents of an aqueous extract of a sample of green propolis from Southeast Brazil were shown by high performance liquid chromatography/mass spectroscopy/mass spectroscopy to be mono- and di-*O*-caffeoylquinic acids; phenylpropanoids known as important constituents of alcohol extracts of green propolis, such as artepillin C and drupanin were also detected in low amounts in the aqueous extract. The anti-inflammatory activity of this extract was evaluated by determination of wound healing parameters. Female Swiss mice were implanted subcutaneously with polyesther-polyurethane sponge discs to induce wound healing responses, and administered orally with green propolis (500 mg kg^−1^). At 4, 7 and 14 days post-implantation, the fibrovascular stroma and deposition of extracellular matrix were evaluated by histopathologic and morphometric analyses. In the propolis-treated group at Days 4 and 7 the inflammatory process in the sponge was reduced in comparison with control. A progressive increase in cell influx and collagen deposition was observed in control and propolis-treated groups during the whole period. However, these effects were attenuated in the propolis-treated group at Days 4 and 7, indicating that key factors of the wound healing process are modulated by propolis constituents.

## 1. Introduction

Propolis is a resinous substance that honeybees produce by mixing their own waxes with resins collected from plants. It is used as a sealant and antimicrobial agent in honeybee nests and has been used as a folk medicine since ancient times. More recently, propolis has been found to have a wide range of biological activities, such as, antimicrobial [1], anti-inflammatory [2], antioxidant [3], hepatoprotective [4], antitumor activities and, in the treatment of dermal lesions such as burns, wounds and ulcers [5–7].

Propolis contains more than 200 constituents, including benzoic acids [8], flavonoids [9, 10] and cinnamic acid derivatives [11]. Depending on the location of production, propolis has a wide variety of botanical origins and the chemical composition varies accordingly. Thus, while caffeic acid phenethyl ester (CAPE) and flavonoids are characteristic constituents of Poplar type propolis [12], the typical constituents of Brazilian green propolis derived from *Baccharis dracunculifolia* are caffeoylquinic and prenylated cinnamic acids, such as artepillin C and baccharin [13, 14]. Other Brazilian green propolis are chemically different; besides prenylated cinnamic acids, they contain triterpenoids [11, 15, 16].

Propolis ethanol extracts (PEE) are most commonly studied in propolis research. In contrast, propolis water extracts (WEP) have rarely been investigated, even though WEP and their main constituents (including caffeoylquinic acids) have higher antioxidant effects, inhibitory activity against some enzymes and absorbency than PEE and their constituents [15]. The constituents of WEP are mainly grouped into two classes, namely, caffeoylquinic acid derivatives (chlorogenic acid, 3,4-di-*O*-caffeoylquinic acid and 3,5-di-*O*-caffeoylquinic acid) and cinnamic acid derivatives (*p*-coumaric acid, baccharin, drupanin and artepillin C) [17], both classes having been shown to exert a variety of biological actions, such as anti-microbial, anti-tumor [5] and antioxidant [2], apoptosis-inducer and immunomodulator properties [6].

Normal wound healing is characterized by inflammatory cell recruitment to the site of injury, invasion of capillary sprouts to the fibrin clot and extracellular matrix components deposition. Several *in vivo* models have been used to characterize various components and mechanisms involved in wound repair and also to assess the effects of potential therapeutic compounds to minimize/improve injury adverse effects. In one such model, the sponge implant, host responses to the synthetic matrix are analogous to the healing process, a complex series of events driven primarily by inflammatory cells that accumulate within the implant compartment. This is followed by angiogenesis and extracellular matrix deposition at the site of injury. The sponge implant model also provides a chronically inflamed environment in which several components of the proliferative fibrovascular tissue (angiogenesis, inflammatory cells recruitment/activation, extracellular matrix deposition) can be determined [18–20].

The present study reports the main constituents of an aqueous extract of Brazilian green propolis from Minas Gerais (Southeast Brazil) and the effects of the orally administered aqueous extract on distinct phases of wound healing (cellular recruitment and extracellular matrix deposition) in a murine sponge model.

## 2. Methods

### 2.1. Preparation of the Aqueous Extract of Green Propolis

Propolis sample was collected in the period of September 2005 to September 2006 in the municipality of Jaguaraçu, Minas Gerais, Brazil from *Apis mellifera* hives. Samples were homogenized and frozen at −18°C and an aliquot (200 g) was powdered and 500 mL of distilled water added. The suspension was maintained for 30–60 min under stirring at 70°C and then cooled at room temperature. The supernatant was filtered through Whatman 1 filter paper to obtain the first extract. The residue was treated again according to the same procedure, with which a second extract was obtained. The two extracts were pooled and then lyophilized.

### 2.2. Analysis of Chemical Composition Using HPLC/DAD/ESI/MS/MS

The lyophilized extract was treated with MeOH:H_2_O (1:1, v/v) at the concentration 1 mg mL^−1^ and filtered through a 0.45-*μ*m filter (German Sciences, Tokyo, Japan). The solution (10 *μ*L) was injected into a DAD SPD-M10AVP SHIMADZU High Pressure Liquid Chromatography (HPLC) system coupled to an ESQUIRE 3000 PLUS, BRUKER DALTONICS mass spectrometer via an electrospray ionization (ESI) source. Spectral UV data from all peaks were accumulated in the range 240–400 nm and chromatograms were recorded at 270 and 340 nm for detection of phenolic compounds. The instrument was controlled by a computer running SCL-10A VP. The mobile phases consisted of eluent A (0.1% aq. HOAc) and eluent B (MeOH) using a reverse phase C18 Zorbax-5B-RP-18 column (4.6 × 250 mm, 5 *μ*m) (Hewlett Packard). A linear gradient of 20%–90% of B (v/v) in the A/B mix was used for analysis, according to the following gradient: 0 min, 20%; 10 min 30%; 20 min, 50%; 30 min, 70%; 40 min, 90%; 45 min, 40%; 50 min, return to the initial condition (20%) to re-equilibrate the column prior to another run. The flow rate was kept constant at 0.5 mL min^−1^ the temperature of the column 28°C. The ionization conditions were adjusted as follows: ESI was achieved using an ion source voltage of −40 V and a capillary offset voltage of 4500 V. Nebulization was aided with a coaxial nitrogen sheath gas provided at a pressure of 27 psi. Desolvation was assisted using a counter current nitrogen flow set at a flux of 7.0 L min^−1^ and capillary temperature of 325°C. The flux of LC/MS was 100 *μ*L min^−1^. ESI/mass spectrometry (MS) parameters, such as nature and flow rate of the sheath liquid, nebulizer pressure, drying gas flow rate and temperature were optimized. MS data were obtained in the positive and negative ion modes. The full scan mass acquisition was performed by scanning from m/z 100–900. Collision-induced fragmentation experiments were performed in the ion trap using He as collision gas, with voltage ramping cycles from 0.3 up to 2 V. The compounds were identified by comparison of their UV and ESI/MS/MS spectra with literature data [21–23]. Relative amounts of constituents in these extracts were assumed to be proportional to the areas under the corresponding chromatogram peaks.

### 2.3. Animals

The 90 female Swiss mice, aged 8 weeks (20–30 g body weight) from the Animal Facility at University Federal of Minas Gerais (UFMG) used in this study were maintained in the animal house of the Department of General Pathology of the Institute of Biological Sciences.

### 2.4. Preparation of the Sponge Discs and Implantation

Polyester-polyurethane sponge discs with 5 mm thickness, 8 mm diameter and weighing 4.6 mg (Vitaform Ltd, Manchester, UK) were used as the matrix for fibrovascular tissue growth. The sponge discs were soaked overnight in a 70% v/v ethanol solution and sterilized by boiling in distilled water for 15 min before the implantation surgery. The animals were anesthetized with a mixture of (10 mg kg^−1^) xylazine and (100 mg kg^−1^) ketamine hydrochloride (i.p.). Their dorsal hair was shaved and the skin wiped with 70% ethanol. Sponge discs were aseptically implanted into a subcutaneous pouch that had been made with curved artery forceps through a 1 cm long dorsal mid-line incision. After sponge implantation, the animals were maintained in individual cages and provided with chow pellets and water *ad libitum*. The light/dark cycle was 12:12 h with lights on at 7:00 am and lights off at 7:00 pm.

Housing and anesthesia concurred with the guidelines established by our local Institutional Animal Welfare Committee (number 161/05).

The treated group (*n* = 8–10) for each time point received propolis orally by gavage (500 mg kg^−1^) daily from the day of implantation and the control group (*n* = 8–10) received saline in the same schedule. At fixed time intervals (4, 7 and 14 days post-implantation) the mice were killed by cervical dislocation and the sponge discs carefully removed, dissected free from the adherent tissue, weighed and processed for histological assessment.

### 2.5. Histology

The sponge implants were carefully collected, fixed in formalin (10% in isotonic saline), embedded in paraffin, and 5 *μ*m-thick sections were obtained. The sections were stained with hematoxylin and eosin for determination of the fibrovascular tissue infiltration and examined under a light microscope. Picrosirius-red staining followed by polarized-light microscopy was used to visualize and determine collagen fibers [24].

### 2.6. Morphometric Analysis of the Fibrovascular Area and Collagen Deposition

Microscopic images of cross-sections (5 *μ*m) were obtained with a planapochromatic objective ×40 (400x) in light microscopy. The images were digitized through a JVC TK-1270/JGB microcamera and transferred to an image analyzer (Kontron Eletronics, Carl Zeiss—KS300 version 2). In order to quantify the fibrovascular area and the deposition of collagen fibers 15 fields were obtained with a planapochromatic objective 10x (100x) for each cross-section and then analyzed morphometrically.

### 2.7. Statistical Analysis

Results are presented as mean  ±  SEM. Comparisons between two groups ( *n* = 8–10 per group) of mice were carried out using Student's *t*-test for unpaired data. A *P*-value <.05 was considered significant. Statistical analysis was performed using Graph-Pad Prism 4.01.

## 3. Results

### 3.1. Chemical Analyses

As shown in Table 1, the major constituents of the aqueous extract of the propolis sample analyzed are caffeoylquinic acids: 4,5-di-*O*-caffeoylquinic (27.2%), 3,4-di-*O*-(*E*)-caffeoylquinic (15.8%), didihydrocaffeoylquinic (13.8%) and 3,5-di-*O*-(*E*)-caffeoylquinic (8.0%). Other caffeoylquinic acids, such as 4-*O*-(*E*)-caffeoylquinic acid, 5-*O*-(*E*)-caffeoylquinic acid and cinnamic acid derivatives, such as artepillin C and drupanin are minor constituents (2.6, 4.4, 5.0 and 2.0%, resp., Table 1).

Peaks 1, 2 and 3, with retention times (RT) 14.4, 17.6 and 20.9, respectively, Table 1, presented m/z 353 in the negative mode, complying with the molecular formula C_16_H_18_O_9_. The corresponding UV spectra are characteristic of caffeoylquinic acid derivatives (UV *≈* 298 and 325 nm). The relative intensities of ions in each spectrum of these chlorogenic acid isomers were significantly different using the same MS/MS condition. MS/MS of 5-*O*-(*E*)-caffeoylquinic acid was dominated by a single peak m/z 191, while MS/MS of 4-*O*-(*E*)-caffeoylquinic acid was dominated by m/z 173 as base peak (100) and, in addition, showed fragments at m/z 179 (50) and 191 (60). MS/MS of 3-*O*-(*E*)-caffeoylquinic acid was dominated by m/z 191 as base peak, accompanied by a fragment at m/z 179 (60). Esters of caffeic with quinic acid at positions 3, 4 and 5 are also known as neochlorogenic, cryptochlorogenic and chlorogenic acid, respectively.

Peaks 5, 6 and 7 (RT 29.0, 31.5 and 32.5, resp., Table 1) showed UV spectra identical with the caffeoylquinic acids described above. Their measured masses were in agreement with masses of protonated ([M + H]^+^) and deprotonated ([M−H]^−^) di-*O*-(*E*)-caffeoylquinic acids, 517.3 and 515.2, respectively, Table 1. The data suggest identity with positional isomers of di-*O*-(*E*)-caffeoylquinic acid, with molecular formula C_25_H_24_O_12_ and molecular weight 516.0. Peaks 5 and 6 were tentatively identified as 3,4-di-*O*-(*E*)-caffeoylquinic and 3,5-di-*O*-(*E*)-caffeoylquinic acid, respectively. As to peak 7, a proposal is put forward that it corresponds to 4,5-di-*O*-(*E*)-caffeoylquinic acid. Base peak of MS/MS spectra of compounds 5–7 is m/z 353, which derives from loss of one caffeoyl fragment; it is thus consistent with the assignment as caffeoylcaffeoyl quinic acids. MS/MS of 3,4-di-*O*-(*E*)-caffeoylquinic acid (peak 5, Table 1) was dominated by m/z 353 (100) and contained fragments at m/z 191 (30), 179 (60) and 173 (25); MS/MS of 3,5-di-*O*-(*E*)-caffeoylquinic acid (peak 6, Table 1) was dominated by m/z 353 (100) and contained fragments at m/z 191 (45), 179 (40) and 173 (20), while MS/MS of 4,5-di-*O*-(*E*)-caffeoylquinic acid (peak 7, Table 1) was dominated by m/z 353 (100) and contained fragments at m/z 191 (20), 179 (20) and 173 (20).

Peak 4 was tentatively identified as a didihydrocaffeoylquinic acid derivative. Its UV spectrum is identical with spectra of caffeoylquinic acids. The compound showed [M−H]^−^ at m/z 519.2 and [M + H]^+^ at m/z 521.4, consistent with molecular formula C_25_H_28_O_12_ and molecular mass 520.0, which corresponds to 4 Da above molecular masses of di-*O*-(*E*)-caffeoylquinic acids.

Peaks 8 and 9 have UV spectra characteristic of cinnamic acid derivatives (315 nm) and [M−H]^−^ at m/z 231.2 and 299.5, respectively, consistent with the known green propolis constituents drupanin and artepillin C, respectively.

### 3.2. Histological Assessments

The sponge matrix was well tolerated by all animals. No sign of infection or rejection was observed in the implant compartment during the 14-day period of the experiment. Systemic administration of propolis (500 mg kg^−1^) during the experimental period (4–14 days) showed no signs of toxicity such as weight loss, sedation, or alterations in motor activity of the animals.

**Figure 1:**
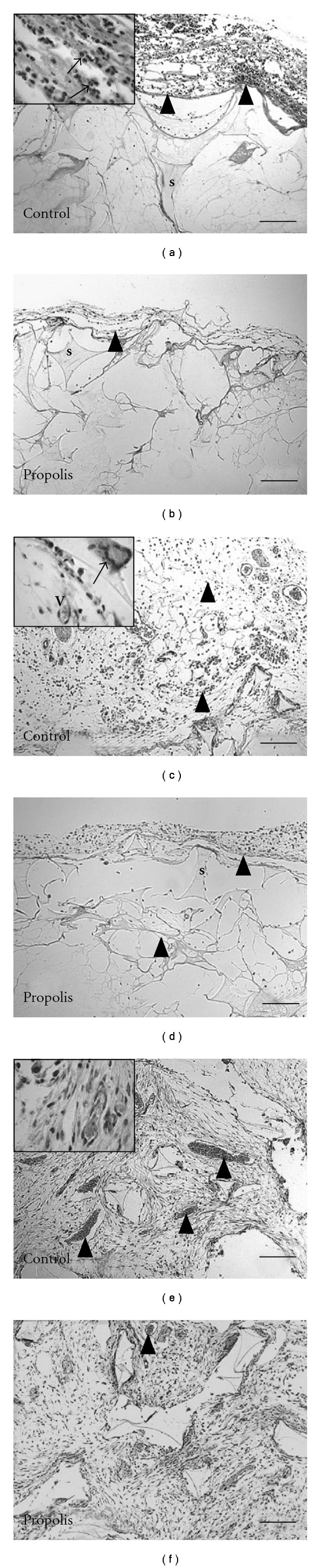
Representative histological sections of sponge implants (5 *μ*m stained with HE) from control (a, c, and e) and propolis-treated groups (b, d, and f) at 4, 7 and 14 days, respectively. The fibrovascular stroma that occupies the pores of the sponge matrix (s) is progressively filled with inflammatory cells, blood vessels (v), fibroblasts and collagen fibers. The granulation tissue in control implants is denser and more vascularized compared with the propolis-treated group. Arrowheads indicate the cell infiltration in the implant (a, b). Inset (1000x): A—arrows show inflammatory cells infiltration; C—arrow shows giant cell and (v) shows blood vessel; E—arrow heads and (v) in the inset (1000x) show blood vessel; F—arrow head shows blood vessel. Bar: 16 *μ*m.

Subcutaneous implantation of sponge discs in mice induced a progressive wound repair response causing the synthetic matrix to be filled with fibrovascular stroma. After HE staining, sponge implants of both groups presented fibrovascular tissue at the three time points studied. The histological changes during the development of the newly formed tissue are illustrated in Figures 1(a)–1(f). At Day 4, the implants in the control group showed much more inflammatory cells accumulation compared with the treated group. Also, in comparison to the treated group, by Day 7 and 14, the sponge in the control group was much more vascularized and contained many more inflammatory cells, multinucleated giant cells and fibroblasts interspersed within the granulation tissue.

### 3.3. Tissue Infiltration and Collagen Deposition

Tissue infiltration and extracellular matrix deposition of the implants were assessed quantitatively from the wet weight of the sponges and morphometric analysis of HE staining sections.

**Figure 2:**
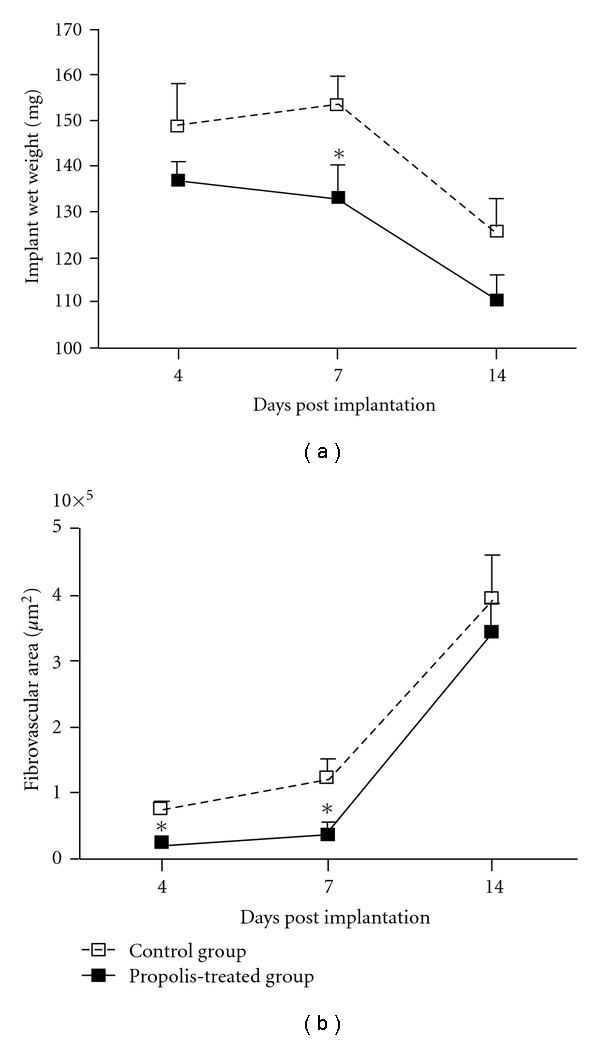
Wet weight (a) and fibrovascular area (b) of the implants from control and propolis-treated groups are shown in this Figure. In the treated group the wet weight was significantly lower than in the control group at day 7. The fibrovascular area in the propolis-treated group was significantly reduced compared with the control group at days 4 and 7. Data are represented as mean  ±  SEM of groups of 8–10 animals. *P < .05 compared with control group.

**Figure 3:**
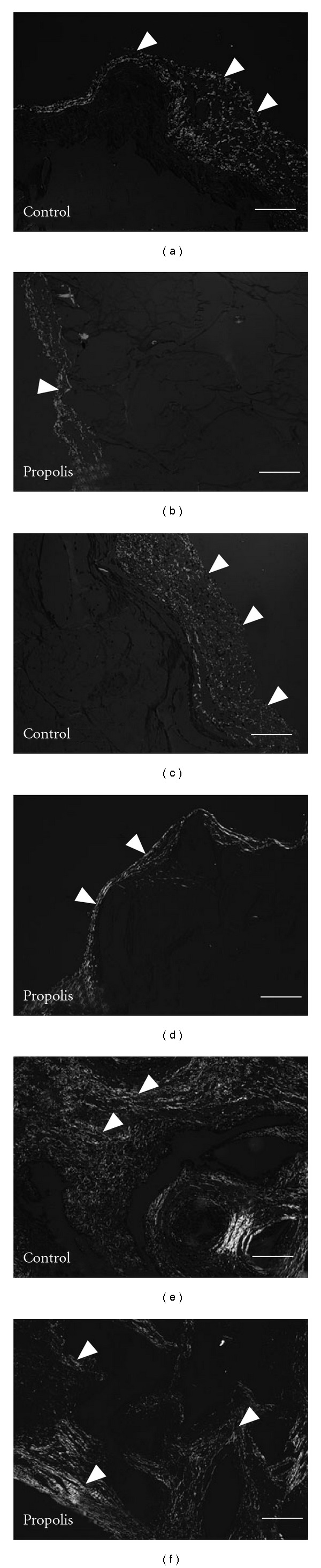
Representative histological sections of sponge implants (5 *μ*m stained with Picrossirius-red) for collagen assessment from control (a, c, and e) and propolis-treated (b, d, and f). The implant was progressively infiltrated by thin collagen (green area) up to 7 days in both groups. After 14 days post implantation, the sponge matrix was predominantly occupied by dense collagen (red and yellow areas) in the propolis-treated and control groups. The collagen area in the propolis-treated group was significantly reduced compared with the control group at days 4, 7 and 14 (arrow heads indicate the most important areas). Bar: 16 *μ*m.

**Figure 4:**
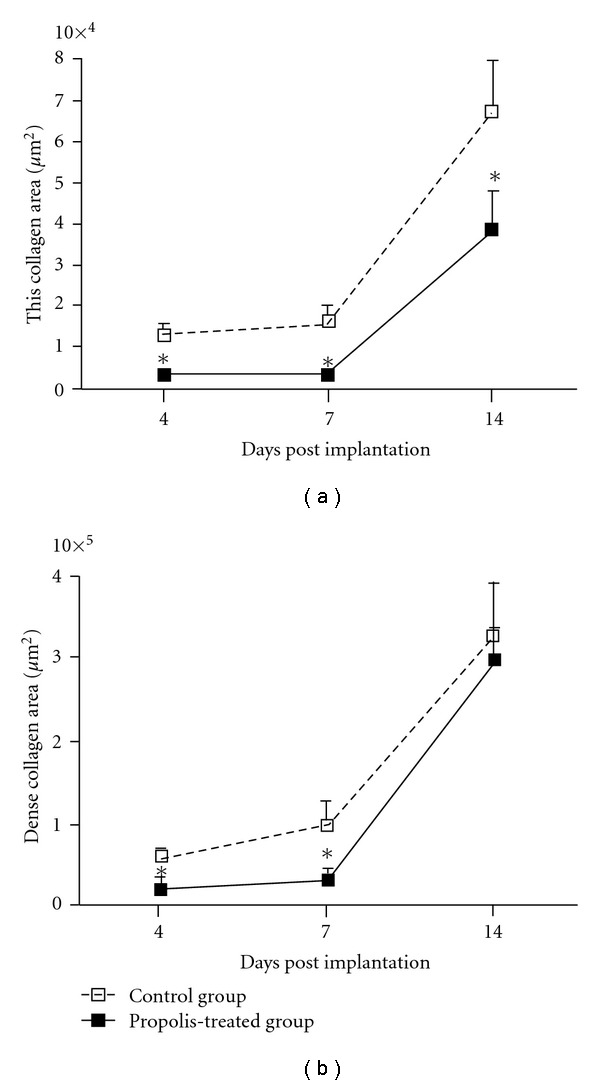
Morphometric analysis of the collagen deposition showing that the deposition of thin (a) and dense (b) collagen in the implants was significantly lower in the treated group at 4 and 7 days post-implantation. Data are represented as mean  ±  SEM of groups of 8–10 animals. *P < .05 compared with control group.

The wet weight of the control implants was significantly higher than the treated implants by day 7 (Figure 2(a). The morphometric area (*μ*m^2^) occupied by the fibrovascular tissue grew throughout the 14-day period postimplantation in both types of implants. However, the fibrovascular area in the control group was bigger compared with the treated group (Figure 2(b)). Both parameters therefore, corroborated the histological assessment (Figures 1(c) and 1(d)). In addition, we investigated the kinetics of collagen deposition during the whole period of sponge implantation as determined by densitometric analysis of implants stained with Picrosirius-red (Figures 3(a)–3(f)). The results clearly show a progressive increase of this extracellular matrix component and an inhibitory effect of propolis in both thin and dense collagen at all time points (Figures 4(a) and 4(b)).

#### 4. Discussion

In this study we determined the chemical composition of a water extract of Brazilian green propolis from Minas Gerais, Brazil [11] and evaluated the systemic effects of this extract in healing parameters in a sponge model. Although propolis is reputed as an anti-inflammatory agent and used to heal a variety of lesions including sores, ulcers and skin incisions [25], its effects on distinct phases of healing processes (cellular recruitment, proliferation and remodeling) has not been evaluated simultaneously in deep skin injury.

Chlorogenic acids are a family of esters containing quinic acid and certain *trans*-cinnamic acids, most commonly caffeic, *p*-coumaric and ferulic. The residues of *trans*-cinnamic acids can be attached at one or more hydroxyls at positions 1, 3, 4 and 5 of quinic acid, originating a series of positional isomers. These compounds predominate in the aqueous extract analyzed and thus are probably the constituents accounting for the observed wound healing effects. Although extensively studied for many years, precise identification of these compounds in plant extracts is difficult due to the lack of commercial standards and the constraints of distinguishing reliably among positional isomers when multiple forms are present at low concentrations and inter-conversion of isomers is possible during workup [26]. Mono- and di-caffeoylquinic acids have been reported as salient compounds of aqueous extracts of Brazilian propolis [13]. The Asteraceae family, which includes *B. dracunculifolia* (source of green propolis resin), is known to be particularly diverse as to the contents of chlorogenic acids. Some but not all of its species are able to esterify all four quinic acid hydroxyls, not just with several cinnamic acids, but also with succinic acid [26].

The implantation technique induced the formation of a fibrovascular stroma that occupied the pores of the sponge matrix with inflammatory cells, blood vessels, fibroblasts and collagen fibers. The granulation tissue in control implants was denser and more vascularized compared with the propolis-treated group. This was reflected in the wet weight of the implants that were lighter in the treated group. Further corroboration of this finding was given by the size of the fibrovascular area that was increased *∼*3-fold in the control group compared with the treated group at Days 4 and 7 post implantation. Our results are in line with the work by others that using different healing models showed decreased cellular recruitment after the treatment with propolis [2, 27–29]. Hu et al. [29] in experiments with carragenin demonstrated that artepilin C was able to inhibit the mobilization of neutrophils in the abdominal cavity. This observation was also recently confirmed by Paulino et al. [2] and in our aqueous extract artepilin C was also detected in a quantity which was probably enough to exert inhibition of inflammatory cell recruitment. It has been reported that phenolic compounds exert a wide range of biological properties such as ability to perform anti-oxidant activity by scavenging free radicals and to accelerate cutaneous wound healing [17, 30, 31].

**Figure 5:**
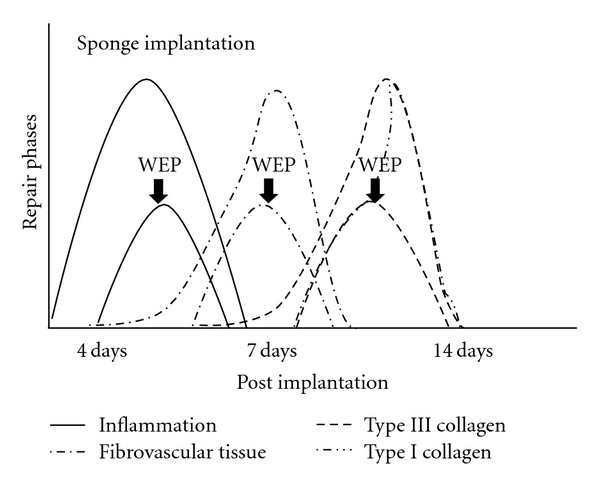
Schematic diagram representing the effects of WEP on fibrovascular tissue growth in sponge implant in mice. WEP attenuated cellular infiltration and deposition of type III collagen, without compromising deposition of type I collagen.

Collagen deposition is an important component of repair. It has been demonstrated that thick fibrils consist of type I collagen and thin, type III collagen [32]. In our experiments, Picrosirius red staining showed two distinct patterns of collagen within the sponge matrix. The deposition of this extracellular component increased progressively from day 4 until day 14 and the fibers became thicker, changing from green (Type III) to yellow/red (Type I) in both types of implants. This profile of collagen deposition in the sponge implants is consistent with previous work in our model as well as in other types of synthetic matrices and in open and incisional wounds [19, 33]. In the propolis-treated group, however, the rate of collagen deposition was delayed at days 4 and 7 postimplantation but reached the levels of the control group by day 14. It is clear that propolis was able to affect collagen type I deposition only at the early phases of the repair process. Probably, attenuation of cell recruitment by propolis has accelerated the proliferative phase of the repair process promoting rapid transformation of type III collagen on type I collagen. Further investigation will be addressed in future experiments to clarify this issue. Our results are in contrast with those by Kilicoglu et al. [34] that showed accelerated healing of colon anastomosis following systemic administration of propolis. One possible explanation for this discrepancy may be due to the intrinsic nature of the tissues evaluated. Another possibility is related with speciogeographic variation of propolis known to determine its chemical composition and biological activities. In addition, the extraction methods (aqueous or ethanolic) constitute another source of variability. Nevertheless, our results show that although propolis has suppressed an important inflammatory component (cell influx) the deposition of collagen at day 14 was not affected by the compound, confirming and expanding the relationship between excess inflammation and aberrant scarring [35]. Thus, our study suggests that aqueous extract of Brazilian green propolis might be used to control the inflammatory response without compromising the repair process as proposed in the hypothetical diagram (Figure 5).

#### Funding

Conselho Nacional de Desenvolvimento Científico e Tecnológico (CNPq) and Fundação de Amparo à Pesquisa do Estado de Minas Gerais (FAPEMIG) in Brazil.

## Figures and Tables

**Table 1:** Chromatographic and spectroscopic data of constituents of an aqueous extract of a sample of Brazilian green propolis from Minas Gerais (Southeast Brazil), obtained by HPLC/ESI/MS

Peak	Retention time (min)	Relative amount (%)	UV (nm)	[M + H]^+^ m/z	[M−H]^−^ m/z	Proposed identification
1	14.4	1.2	ND	355.2	353.4	3-*O*-(*E*)-caffeoylquinic acid
2	17.6	4.4	300 sh, 330	355.2	353.1	5-*O*-(*E*)-caffeoylquinic acid
3	20.9	2.6	300 sh, 330	355.2	353.1	4-*O*-(*E*)-caffeoylquinic acid
4	27.1	13.8	300 sh, 330	521.4	519.2	Didihydrocaffeoylquinic acid
5	29.0	15.8	300 sh, 330	517.3	515.2	3,4-Di-*O*-(*E*)-caffeoylquinic acid
6	31.5	8.0	300 sh, 330	517.3	515.2	3,5-Di-*O*-(*E*)-caffeoylquinic acid
7	32.5	27.2	330	ND	515.2	4,5-Di-*O*-(*E*)-caffeoylquinic acid
8	41.3	2.0	315	ND	231.2	4-Hydroxy-3-prenyl-cinnamic acid (drupanin)
9	47.0	5.0	315	ND	299.5	4-Hydroxy-3,5-diprenyl-cinnamic acid (artepilin C)

ND: not determined.
